# Changes in RNA Splicing in Developing Soybean (*Glycine max*) Embryos

**DOI:** 10.3390/biology2041311

**Published:** 2013-11-21

**Authors:** Delasa Aghamirzaie, Mahdi Nabiyouni, Yihui Fang, Curtis Klumas, Lenwood S. Heath, Ruth Grene, Eva Collakova

**Affiliations:** 1 Genetics, Bioinformatics and Computational Biology Program, Virginia Tech, Blacksburg, VA 24061, USA; E-Mails: delasa@vt.edu (D.A.); curtisk@vt.edu (C.K.); 2 Department of Computer Science, Virginia Tech, Blacksburg, VA 24061, USA; E-Mails: nabiyoun@vt.edu (M.N.); heath@vt.edu (L.S.H.); 3 Department of Plant Pathology, Physiology, and Weed Science, Virginia Tech, Blacksburg, VA 24061, USA; E-Mails: rainfyh@vt.edu (Y.F.); grene@vt.edu (R.G.)

**Keywords:** abscisic acid, alternative splicing, auxin, central carbon and nitrogen metabolism, desiccation tolerance, dormancy induction, post-transcriptional regulation, seed and embryo development, soybean

## Abstract

Developing soybean seeds accumulate oils, proteins, and carbohydrates that are used as oxidizable substrates providing metabolic precursors and energy during seed germination. The accumulation of these storage compounds in developing seeds is highly regulated at multiple levels, including at transcriptional and post-transcriptional regulation. RNA sequencing was used to provide comprehensive information about transcriptional and post-transcriptional events that take place in developing soybean embryos. Bioinformatics analyses lead to the identification of different classes of alternatively spliced isoforms and corresponding changes in their levels on a global scale during soybean embryo development. Alternative splicing was associated with transcripts involved in various metabolic and developmental processes, including central carbon and nitrogen metabolism, induction of maturation and dormancy, and splicing itself. Detailed examination of selected RNA isoforms revealed alterations in individual domains that could result in changes in subcellular localization of the resulting proteins, protein-protein and enzyme-substrate interactions, and regulation of protein activities. Different isoforms may play an important role in regulating developmental and metabolic processes occurring at different stages in developing oilseed embryos.

## 1. Introduction

Seed filling, the induction of dormancy, and the acquisition of desiccation tolerance constitute essential events in soybean seed development. Seed storage compounds (oil, protein, and carbohydrates) accumulating during seed filling provide substrates and energy in germinating, photosynthetically incompetent seedlings [1,2,3]. In soybean, seed storage compounds are synthesized through pathways of central carbon and nitrogen metabolism (CCNM) [4,5,6,7,8,9]. Drying seeds initiate molecular and physiological responses leading to dormancy and the acquisition of desiccation tolerance, ensuring seed viability during storage preceding germination [10,11,12,13]. The processes that occur during the various stages of embryo and seed development and maturation are highly regulated at multiple levels. Post-transcriptional regulation represents one of the many tiers of complex regulatory events accompanying embryo and seed development and metabolism.

Alternative splicing (AS) is a post-transcriptional regulatory process contributing to transcriptome and proteome diversities by enabling the production of multiple mRNA and protein molecules from a single gene [14,15]. Different splice isoforms, originating from the same gene, may contain or lack specific sequences, including functional, regulatory, and interaction domains, as well as organelle localization sequences. As such, the resulting mRNA and protein molecules may be affected in terms of stability, subcellular localization, structure, protein-molecule interactions, regulation, and function [16,17]. Plants contain numerous non-consensus splice sites in their genomes that tend to lead to transcripts with premature protein synthesis termination [18,19]. Some truncated mRNA molecules are subjected to nonsense-mediated decay (NMD), a mechanism proposed to regulate transcript abundance [20,21,22]. In *Arabidopsis thaliana*, approximately 13% of transcripts containing introns are templates for truncated mRNA molecules degraded by NMD and were shown to play major roles in development, regulation, and stress responses [21]. 

In plants, AS is a widespread phenomenon, as between 20% to 60% of plant genes encode splice variants in different plant species and under different conditions [17,18,23,24]. AS is involved in regulating expression of plant genes involved in various aspects of growth and development [20,25,26,27,28,29,30], and responses to environment including abiotic and biotic stresses [31,32,33,34,35,36,37]. The importance of AS in plant metabolism has not been as well-documented as for development-, growth-, and stress-related processes. Nevertheless, AS plays a role in many aspects of plant metabolism, including regulating protein stability [38], enzyme activity [39,40], sub-cellular localization of enzymes [41,42,43,44,45], and metabolic responses to stress [46].

AS also plays an important role during early [47] and late [48] phases of seed and embryo development. AS of ABI3 (ABSCISIC ACID INSENSITIVE 3), a transcriptional regulator of seed development in Arabidopsis, is regulated by the splicing factor SUA (SUPPRESSOR OF ABI3) [48]. In Arabidopsis, the phytochrome-interacting factor PIF6 is a transcription factor (TF) existing as two splice isoforms [49]. The full length variant has a potential DNA-binding domain that is spliced out of the short variant, resulting in the introduction of a stop codon. Overexpression of the short, but not the long isoform reduces seed dormancy [49]. Acquisition of desiccation tolerance in maturing seeds shares similarities with abiotic stress responses in plants [10,50,51,52,53]. It is reasonable to expect that some AS events regulating seed maturation will be conserved between these processes.

Here, we present results from a comprehensive global AS analysis of the transcriptome in developing soybean embryos during seed filling and maturation. Our results indicate that AS is a frequently occurring phenomenon in both the metabolic and the hormone-mediated signaling processes that occur during seed filling and acquisition of dormancy and desiccation tolerance in developing soybean embryos. We discuss these findings from a global perspective as well as by focusing on selected examples of AS-derived protein isoforms involved in various aspects of CCNM and ABA- and auxin-mediated signaling relevant to the maturation and desiccation processes.

## 2. Experimental Section

### 2.1. RNA-Sequencing-Based Transcriptomics

**Figure 1:**
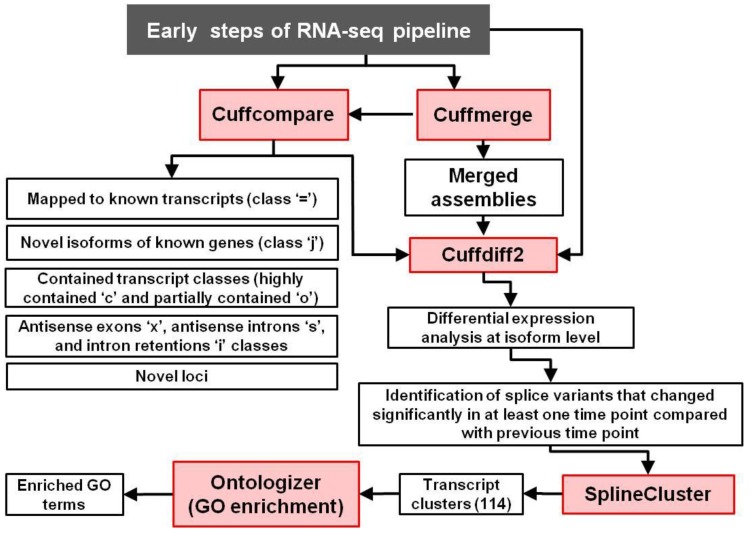
Flowchart of bioinformatics analyses used for differential expression of splice isoforms and subsequent data mining. The initial steps of the RNA-seq pipeline are described in [53] and tools are in red boxes.

In the previous study, a detailed time-course of soybean embryo development, involving ten time points with three replicates each, was performed [53]. Reads were mapped to the Gmax_109 version of the soybean *G. max* (cv. Williams 82) genome, which was recently sequenced [54] and subjected to an RNA-seq and differential gene expression analyses pipeline as described [53]. The resulting data sets are available in the Gene Expression Omnibus database (GEO accession number GSE46153). For this study, the newest available version of the genome from Phytozome (Gmax_189 [55,56]) was used for analyzing the transcripts reported here. Briefly, the Tuxedo Suite-based RNA-seq analysis pipeline consists of the following steps. First, the reads are mapped to the reference genome using Tophat [57]. Second, the reads are concatenated using Cufflinks [58] and the RABT (Reference Annotation Based Transcript) assembly technique is used for this purpose [59]. This results in a good accuracy for finding novel genes and splice isoforms when high-quality sequence information exists for that genome. The assembled transcripts from all samples are merged using Cuffmerge and are compared with the reference genome using the Cuffcompare tool to find known and novel genes and isoforms, as well as transcripts expressed from intergenic regions. Third, the reads from TopHat and merged assemblies from Cuffmerge are used as an input for Cuffdiff2 [60]. The GTF annotation file resulting from Cuffmerge analysis and containing merged annotation of all assembled transcripts is provided in the supplementary data as the “merged.gtf” file. Cuffdiff2 in the time course mode is then used for differential expression analysis of individual transcripts within the RNA-seq data and the bioinformatics analysis pipeline is presented in Figure 1. 

Cuffdiff2 is an excellent isoform-based differential expression analysis tool [60]. We also explored two other leading tools for further AS analysis. SpliceGrapher is an isoform-based AS analysis tool thought to be superior to Cuffdiff2 as it minimizes the identification of false positives [61]. However, closer examination revealed that SpliceGrapher does not consider the non-canonical splice sites that are frequently found in plants [18,19] and the isoforms resulting from splicing at those sites are considered false positives by the tool. As such, SpliceGrapher is limited to the identification of known plant transcripts without the potential of retrieving novel transcripts originating from non-canonical splicing. Unlike Cuffdiff2 and SpliceGrapher, DEXSeq is an exon-based tool for AS analysis and it was not used because it is intended for differential expression of known individual exons and introns rather than whole transcripts [62]. Accordingly, Cuffdiff2 [60] was further used for differential expression analysis of transcripts in developing soybean embryos, while SpliceGrapher [61] was used to visualize selected isoforms based on existing gene models and Cuffdiff2 data due to its superior graphical isoform representation. 

Cuffdiff uses a set of 12 class codes assigned in Cuffcompare to categorize assembled transcripts obtained from Cufflinks [58,63]. Briefly, these class codes serve as a basis for information about the structure of the various assembled transcripts with reference to transcripts with well-characterized splicing patterns (class “=”). It is noteworthy that assignment of class codes in Cuffdiff is prioritized. For example, when an isoform has a novel splice junction it is classified as class “j”, although its structure may fall into other lower priority classes as well. Class “j” transcripts are potentially novel isoforms, in that they have at least one novel splice junction and at least one splice junction shared with the reference transcript. Class “o” transcripts are assembled transcripts that show exonic overlap with the reference transcript, but do not fall into other higher priority class such as “c” or “j”. Class “c” stands for “contained” and is used when a transcript has a high exonic overlap with a known transcript. Class “c” was not observed in our significantly differentially expressed transcripts, but has a high priority among class codes. Transcripts in classes “x” and “s” have exonic and intronic overlap, respectively, with the reference transcript on the opposite (antisense) strand. Class “i” transcripts are those, for which some sequence falls entirely within a reference intron. These transcripts are representative of intron retention events, in which the transcript did not fall into other higher priority classes. We named new assembled transcripts based on the number of known transcripts for each gene entered in the Gmax_189 version of the soybean reference genome. For example, if the number of known transcripts for the gene X is 2 (X.1 and X.2) then the first novel transcript will be designated X.N3. 

### 2.2. AS and Clustering Analyses

For further investigation of the 13,253 differentially expressed transcripts, we clustered them using SplineCluster [64]. SplineCluster is a model-based hierarchical co-clustering tool for analyzing gene expression data fitting statistical regression models to a gene expression time series data. Therefore, SplineCluster can be used to group the transcripts with a common expression pattern over time [64]. Because we were interested in the changing behavior of alternatively spliced transcripts over time during soybean embryo development rather than actual expression values, the FPKM values originating from Cuffdiff2 were scaled to have an arbitrary mean of 10,000. The resulting expression values were clustered using SplineCluster, which yielded 114 clusters with the prior precision of 10^−10^ and 10^13^ reallocation sweeps (number of iterative reclassifications). In order to further group the genes in each cluster based on their functional description, we carried out Gene Ontology (GO) enrichment analysis using Ontologizer [65]. The Topology Elim option, in conjunction with Westfall-Young Single Step were used for GO enrichment analysis with 10,000 re-sampling steps (number of steps used in re-sampling-based corrections for multiple testing). GO enriched terms with a *p*-value < 0.05 were identified as statistically significant and were subjected to further data mining.

We were interested in assessing how statistically significant (*p* < 0.05) enriched GO terms related to “RNA splicing”, “dormancy”, and “response to hormone stimulus”, especially ABA and auxin, were distributed across the GO tree. Therefore, the enriched GO terms related to these three keywords were filtered among the GO enrichment results of all 114 clusters. The inferred tree and the list of ancestors and children for each enriched GO term of interest were obtained from the GO database [66] and then were combined to get a single tree to cover all enriched GO terms corresponding to each keyword. This tree was then split for visualization purposes to represent three individual sub-trees relevant to the three categories of interest separately. 

### 2.3. Quantitative Real-Time PCR (qPCR) Validation of Gene and Isoform Transcriptional Changes

Thirteen genes and eight splice isoforms representing four genes (Table S1) were selected to validate the observed RNA-seq-based gene and isoform abundance changes by qPCR at time points of interest. Specifically, time points at which transcript levels changed significantly compared to the previous time point were used. Selection of genes and isoforms for validation was based on previously [53], and currently, analyzed data and several criteria in order to capture: (i) distinct expression patterns; (ii) relative abundance levels (low, intermediate, and high, based on normalized FPKM values); and (iii) documented functions in metabolism, dormancy induction, or the acquisition of desiccation tolerance. Two genes (Glyma14g00360 and Glyma13g16500) that showed stable expression, but different abundances (moderately high and low) were used as controls. One of them (Glyma14g00360) was used as an internal control for qPCR normalization. The other gene (Glyma13g16500) showed very low and inconsistent expression with qPCR (C_T_ > 32 in a 40-cycle amplification program). Low and inconsistent Glyma13g16500 transcript levels were observed also for the second (backup) primer pair. As such, Glyma13g16500 transcript levels could not be used for normalization of transcript levels. Specific primer pairs were designed to amplify common transcript regions for selected genes as well as unique regions of each selected isoform (Table S1).

Total RNA was isolated as described previously [53]. cDNA was prepared by using TaqMan reverse transcription reagents and MultiScribe reverse transcriptase according to the manufacturer’s protocols (Invitrogen, Carlsbad, CA, USA). qPCR was performed by using SYBR Green PCR Master Mix (Applied Biosystems, Foster City, CA, USA) according to the manufacturer’s recommendation on an ABI 7500 Series RT-PCR System (Applied Biosystems). Three biological replicate reactions along with one non-template control were performed for each sample. The 2^−∆∆CT^ method was used to determine the relative transcript levels of genes and isoforms [67]. Briefly, qPCR transcript data were first normalized to that of the internal control (Glyma14g00360) in every sample. These normalized values were then compared to these in samples involving the first selected time point to obtain relative amount of transcript levels. Results of this analysis are presented in Figure S1. In general, trends obtained with RNA-seq were consistent with qPCR, with the exception of Glyma08g24420 (WRINKLED1) that showed a very low or non-existent expression with qPCR (C_T_ > 35). In Arabidopsis, the *WRINKLED1* gene is known to be expressed prior to oil accumulation, followed by a decrease during the early stages of seed filling [68], which is consistent with our RNA-seq data.

## 3. Results and Discussion

In our previous study, data related to global temporal transcriptional and metabolic changes were discussed in the context of seed filling and maturation processes leading to accumulation of seed storage compounds, dormancy induction, and acquisition of desiccation tolerance in developing soybean embryos [53]. The developmental time course of soybean embryos involved time points relevant to these two major processes, starting with 3-mm long, light-green photoheterotrophic embryos that had already started to accumulate storage oil and proteins (17- to 22-day-old fully differentiated embryos representing time point 0). Embryos were collected at five-day intervals to obtain a detailed time course (time points 1 through 9 corresponding to days 5 through 45, respectively), except for the last time point (day 55) having a 10-day interval. Embryos at the last time point were already yellow and incapable of photosynthesis. This detailed time course enabled the capture of transcriptional and post-transcriptional events underlying both the seed filling and early maturation phases of soybean embryo development [53]. Here, we use these detailed data sets to assess global AS in seed filling and maturation stages of soybean embryo development and to target genes involved in CCNM, dormancy induction, and acquisition of desiccation tolerance.

### 3.1. Global Assessment of AS in Developing Soybean Embryos

**Figure 2:**
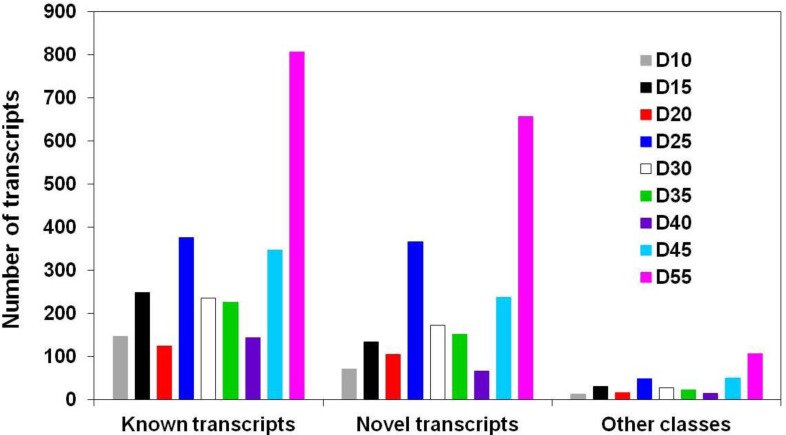
Distribution of known, novel, and other class isoforms in developing soybean embryos. Results are shown for 2,942 isoforms encoded by genes expressing between two and six isoforms showing changes in their transcript levels during soybean embryo development (day 10 (D10)–day 55 (D55)). Other Cuffcompare classes represent o (partial overlap with known transcript), x (antisense exon), and s (antisense intron).

The soybean genome contains 54,175 genes and 73,320 known transcripts to date, suggesting that, on average, a single gene encodes about 1.35 transcripts [56,69]. In Arabidopsis, this number is 1.29 based on the TAIR10 gene annotation data [70], suggesting that the frequency of AS in Arabidopsis is similar to that of soybean, provided that similar types of tissues and conditions were tested in these two plant species for transcriptomics. It is clear that some genes only encode a single transcript, while others encode multiple transcripts, and we first aimed to assess the extent of AS in developing soybean embryos. Overall, 47,331 genes were expressed in developing soybean embryos, giving rise to 217,371 total transcripts (many of them novel) and, on average, over 4.6 transcripts per gene. This is a larger number than anticipated based on the existing soybean genome data. However, AS events, which occur transcriptome-wide during soybean embryo development, have not been studied in detail before. Desiccation-related processes similar to drought and salt stress responses are involved, and AS is known to be induced as a result of stress imposition [31,32,33,34,35,36]. In fact, the majority of AS events took place during the late maturation and desiccation stages of soybean embryo development. Nearly 50% of differentially expressed transcripts showed an increase in abundance in the last 2–3 time points. Most importantly, the frequency of 1,395 differentially expressed genes that had two or more transcripts showing changes in abundances at least in one time point (Table 1) was the highest at the last time point (Figure 2), suggesting that AS was especially induced during embryo maturation, dormancy and desiccation tolerance acquisition stages.

**Table 1:** Transcripts showing altered levels relative to the previous time point. The total number of genes that had significant transcriptional changes for at least one of their isoforms was 15,368, while the number of genes with more than one differentially expressed transcript was 1,395. The total number of isoforms for multi-transcript genes showing differential expression was 2,942. The level of significance for differential expression was set by using the *p*-value cutoff of 0.05.

Number of genes	13,973	1,270	102	20	2	1
Number of transcripts/isoforms	1	2	3	4	5	6

The total number of isoforms (encoded by 15,368 genes) that showed changes in their transcript levels during embryo development was 16,915 (*p*-value < 0.05 and false discovery rate (FDR) < 5%). Out of these 15,368 genes, 13,973 genes each expressed a single isoform exhibiting statistically significant (*p* < 0.05) changes in their transcript levels in at least one time point in developing soybean embryos (Table 1). Each of the remaining 1,395 genes had between two and six isoforms showing changes in their transcript levels (Table 1). Among these 13,973 genes, 10,311 genes also had each at least one other, stably expressed isoform in addition to the isoform showing developmental transcript pattern changes (Table 2). As these isoforms showed different expression patterns (for each of 10,311 genes, one isoform displayed changes in its transcript levels, while the other(s) did not), they were considered to be differentially expressed relative to each other. As such, a total of 11,706 (10,311 and 1,395) genes that were represented by at least two differentially expressed isoforms were found to be alternatively spliced. Only 3,662 out of 13,973 genes were represented by a single transcript each. Many of these transcripts are known, and appear to be a result of conventional splicing rather than AS, as only 15 belong to one of the other Cuffcompare [58,63] classes (j, i, and o, in Table 2).

**Table 2:** Distribution of isoforms among Cuffcompare classes. The 16,915 transcripts that showed significantly (*p*-value < 0.05, FDR < 5%) changed levels during embryo development, originating from 15,368 genes are classified below. Cuffcompare codes and descriptions are shown on the left for isoforms originating from three different sets of genes. The first set of genes (10,311) encodes single isoforms showing significant changes in developing soybean embryos. At least one additional, stably expressed isoform (not included in the data presented in this table) is also produced from this set of genes. Each of the second set of genes (3,662) corresponds to only a single isoform showing developmental changes in transcript levels in soybean embryos. The third set of genes (1,395) corresponds to at least two differentially expressed isoforms.

		One transcript changed and at least one other did not change	Single transcript	At least two isoforms changed
	Genes	10,311	3,662	1,395
**Codes**	**Corresponding isoforms that showed differential expression**			
=	Known transcript	8,466	3,647	1,421
j	Novel transcript	1,525	12	1,313
i	Intron retention	3	1	0
o	Partial overlap with known reference transcript	183	2	68
x	Antisense exon	128	0	116
s	Antisense intron	6	0	24
	Total	10,311	3,662	2,942

Our significantly differentially expressed transcripts fall into six Cuffcompare classes (=, j, o, x, s, and i) [58,63]. The majority (75%) of these transcripts were previously known (= class). The remaining 25% are divided among the other classes listed (Table 2). 10,311 genes that had one isoform changing significantly along with at least one stably expressed isoform encoded mostly known transcripts (82%), while only 15% of novel isoforms, (j class), were observed (Table 2). Only 3% of these transcripts belonged to one of the other four Cuffcompare classes represented in our dataset (i, o, x, and s). For 1395 genes that had more than one differentially expressed transcript, known and novel isoforms were about equally frequent and represented the majority of transcripts, while other classes had only 7% representation (Table 2). This is not unexpected, as only one or two known isoforms have been reported for most soybean genes [69,71]. As such, genes encoding more than one isoform are by definition enriched in this group. Collectively, these observations suggest that the majority of genes showing changes in transcript levels in developing soybean embryos produce multiple transcripts due to AS.

### 3.2. Differential Expression of Related Isoforms Involved in CCNM and Maturation during Soybean Embryo Development

While AS plays major roles in regulating a variety of growth-, development-, and stress-related processes, its role in regulating metabolism has so far been neglected. Accumulation of seed storage compounds and the underlying metabolism leading to the synthesis of these compounds as well as processes relevant to maturation and dormancy represent important stages in seed development. Expression of some of the genes involved in these processes could be regulated by AS. Our goal was to estimate the extent of AS events in developing soybean embryos, with the focus on genes encoding enzymes and proteins involved in CCNM and acquisition of desiccation tolerance. In the previous study, we identified expression patterns of these genes [53], while here we aimed to address the question of whether any of these genes were subjected to AS.

First, co-expression analysis was performed on 15,368 transcripts displaying changes in their abundance in a time-series-dependent manner using the Bayesian co-clustering SplineCluster tool [64]. This analysis yielded 114 clusters (Figure S2). Searchable information about individual isoforms and clusters is available in Table S2. Each transcript showed a defined temporal expression pattern and belonged to one of 114 clusters representing those expression profiles. Clusters representing temporal trends characteristic of previously identified CCNM- and maturation-related genes [53] were comprehensively mined for the processes and genes of interest by using GO enrichment strategies.

Although every cluster was closely examined, certain patterns were of particular interest and involved three major trends: (i) an initial sharp decrease followed by undetectable, or stable low, transcript levels found for genes encoding CCNM enzymes supporting cell division; (ii) a gradual increase followed by a gradual decrease in transcript levels, as exemplified for seed filling CCNM genes; and (iii) undetectable or very low initial stable transcript levels followed by a sharp increase in transcript abundance in the last two or three time points observed for genes encoding proteins involved in maturation processes. Since ABA-related processes are crucial for dormancy and desiccation tolerance acquisition [72], as well as the action of other hormones, such as auxin and jasmonic acid [73], splicing events in those categories were also queried. Genes associated with splicing itself were examined as well, as there is evidence that these transcripts are often the product of stress-mediated splicing themselves [74], giving rise to specific isoforms that have the potential for roles in regulation.

#### 3.2.1. Central Carbon and Nitrogen Metabolism

The majority of isoforms showing changes in abundances were associated with various largely unknown cellular and regulatory processes and only a small proportion of these transcripts were encoded by metabolic genes. This is in agreement with the observation that genes encoding CCNM enzymes represent only a small proportion of the total genes in plant genomes [75,76,77,78]. We previously identified two different sets of homologous CCNM genes involved in two different types of metabolism proposed to support: (i) cell division in differentiating and young embryos, and (ii) cell elongation and accumulation of seed storage compounds during seed filling [53]. The question is whether the pre-mRNA molecules originating from these different sets of genes are subjected to AS and whether the resulting isoforms also show distinct expression patterns.

**Figure 3:**
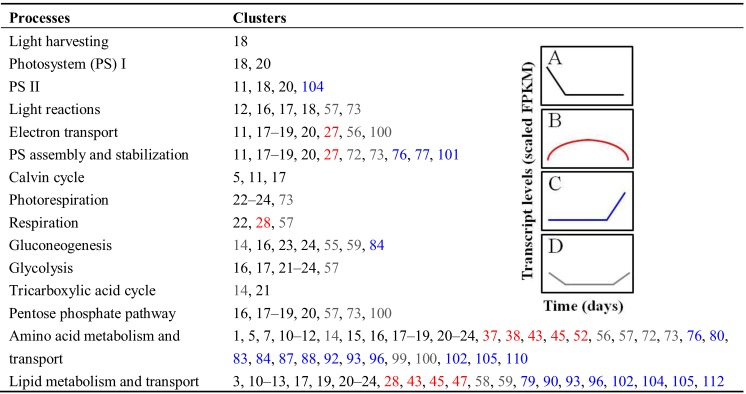
Major metabolic processes of CCNM and relevant clusters enriched in transcripts encoding a variety of proteins involved in these processes. GO enrichment analysis was performed on isoforms in all 114 clusters (*p*-value < 0.05). Only clusters displaying four basic trends consistent with: (**A**) early (initial decrease followed by stable low or no expression; black); (**B**) seed filling (moderate increase followed by a moderate decrease; red); (**C**) maturation (stable low or no initial expression followed by a final increase; blue); and (**D**) early and maturation (initial decrease followed by no or low stable expression and final increase in transcript levels; gray) CCNM are shown. Clusters showing conceptually similar trends based on visual assessment have the same color that matches the corresponding trend.

Every cluster contained some metabolic genes (Table S2), but only some clusters displaying specific expression patterns were enriched with many alternatively spliced transcripts encoding different enzymes and metabolite transporters involved in CCNM pathways including photosynthesis, photorespiration, respiration, glycolysis, gluconeogenesis, tricarboxylic acid (TCA) cycle, pentose phosphate pathway, and amino acid and lipid metabolism. Results regarding GO enrichment in specific metabolic processes are summarized in brief in Figure 3 and in detail in Table S3. Clusters 1–26 (black in Figure 3) were consistent with the gene expression patterns of CCNM genes predicted to support cell division and differentiation in young embryos. This trend is characterized by an initial high transcript levels at day five followed by decreases of variable slopes until day 15 or 25 and a subsequent stable very low or no expression for the remainder of the developmental time course. Isoforms present in cluster 14 also showed a moderate increase in abundances from day 40 to day 55, suggesting that they are also involved in CCNM supporting maturing and desiccating embryos.

We identified other clusters showing trends that resembled the expression pattern of cluster 14, but exhibited subtle initial decreases and quite considerable final increases in expression patterns (clusters 55–59, 72, 73, and 99–100; shown in gray in Figure 3). These clusters (including cluster 14) were enriched in transcripts encoding enzymes and transporters involved in photosynthesis, photorespiration, gluconeogenesis, glycolysis, the TCA cycle, respiration, and amino acid and lipid metabolism. Many of these processes, particularly gluconeogenesis, glycolysis, the TCA cycle, respiration, and amino acid metabolism are coupled with oil degradation occurring during the maturation of oilseeds. These processes provide substrates and energy for growth and continuing seed storage protein accumulation in drying seeds when the connection between the maternal tissue and the seed is gradually severed [79,80].

However, it is also possible that some of these alternatively spliced transcripts accumulate and are stored in RNA-processing bodies (P-bodies) or stress granules in drying embryos [81,82,83,84]. These transcripts encode proteins and enzymes involved in seed storage compound mobilization during germination, which involves similar types of metabolic processes as does heterotrophic metabolism during late maturation phases of embryo development [1,2,3,79,80]. In addition, photosynthetic activity is absent in yellow drying embryos and photosynthesis-related transcripts accumulating in these embryos could be stored for mobilization during and after germination. Storing these transcripts, along with transcripts encoding enzymes involved in the mobilization of storage lipids, proteins, and carbohydrates, is advantageous for germinating seeds and their vigor [85]. It appears that AS events taking place during embryo development also play a role in germinating seeds. 

Such surprising, but not entirely unexpected, potential multiple functions in different types of CCNM for these isoforms remain to be confirmed. Nevertheless, the use of the same transcripts for CCNM at completely different stages of plant development (early and late stages of seed development as well as seed germination) suggests existence of common regulatory components of AS and subsequently CCNM during these different developmental phases.

Clusters 27, 28, 37, 38, 43, 45, 47, and 52 (red in Figure 3) share a common overall trend of an initial moderate increase from day 5 to 20 or 25, followed by a gradual moderate decrease in isoform transcript abundance (Figure S2). This trend is consistent with the trend in the transcription of CCNM genes potentially involved in seed filling [53], but only specific processes and fewer genes are represented here than in the case of the potential cell division CCNM genes. In the case of photosynthesis and respiration, transcripts encoding several light-harvesting complexes and electron carriers are present in these clusters. In contrast, amino acid and lipid metabolism and transport GO categories dominate these clusters from the perspective of metabolism, however, they are underrepresented relative to cell division-related CCNM (Table 3, Table S2 and Table S3).

The ACT (Asp kinase, Chorismate mutase, and TyrA) domain enables binding of amino acids to proteins and subsequently allosteric regulation of enzyme activity [86,87,88]. The Arabidopsis genome contains at least 12 copies of these genes that can have functions as sensors or in the regulation of metabolism [89,90]. Interestingly, ACT domain-containing proteins represented by the transcripts Glyma10g42580.N6, Glyma18g52120.1, Glyma13g09310.4, and Glyma15g00560.N7 were also identified among AS-derived isoforms belonging to the seed filling clusters (Table S2 and Table S3). Glyma10g42580 (ACR12 in Arabidopsis) was expressed as two isoforms (5 and N6) exhibiting completely different expression patterns (clusters 23 and 37, respectively), while the rest of these ACR genes had at least one other stably expressed transcript. 

**Table 3:** Isoforms present in clusters 27, 28, 37, 38, 43, 45, 47, and 52 representing central carbon and nitrogen metabolism (CCNM) supporting seed filling. Amino acid and lipid metabolism is dominating these clusters.

Process	Isoforms	Annotation
Photosynthesis	Glyma05g24660.N2; Glyma18g03220.3; Glyma02g47560.2; Glyma20g35530.1	Light-harvesting complexes
	Glyma07g21150.N2; Glyma11g08230.1; Glyma05g03730.1; Glyma12g32680.1	Electron carriers
	Glyma15g40450.1	O_2_-evolving complex
Respiration	Glyma10g08480.1	Kinesin-like protein 1
	Glyma08g00880.N4	NADPH/respiratory burst oxidase protein D
Amino acid metabolism	Glyma19g27500.1; Glyma11g38130.1; Glyma18g06840.N6; Glyma06g13280.N3; Glyma14g32500.1; Glyma19g28770.1	Asp- and Glu-family enzymes
	Glyma15g05630.1	Ser decarboxylase
	Glyma10g35580.N13; Glyma12g07720.N10	Aromatic amino acid enzymes
	Glyma04g42190.1; Glyma07g30500.N8; Glyma19g29880.N2	Branched-chain amino acid enzymes
	Glyma13g24580.N25; Glyma02g42800.1; Glyma09g37270.N2	Amino acid transporters
Lipid metabolism	Glyma05g24650.N3; Glyma17g07480.N2; Glyma05g36910.1; Glyma12g13020.1; Glyma07g18370.N4; Glyma06g17640.1; Glyma17g03070.N3	Acyl-CoA-related enzymes
	Glyma18g06950.1; Glyma14g37350.N3	Fatty acid desaturases

Numerous clusters showing low, but stable, or undetectable transcript levels from day 5 to day 35–45, followed by a substantial transcript abundance increase of varying slopes (Figure S2, relevant clusters are highlighted in blue in Figure 3) were enriched in a large number of isoforms involved primarily in CCNM and transport involving amino acid and lipid metabolism (Figure 3, Table S2 and Table S3). This is expected, as during the late developmental stages, embryos degrade their chlorophyll through ABA-mediated signaling and photosynthesis ceases to function [91,92]. This means that these embryos rely on nutrients of maternal origin as well as on internal lipid degradation to provide carbon and energy sources for ongoing seed storage protein accumulation and metabolism relevant to maturation and acquisition of desiccation tolerance [79,80,93]. Because of the large number of transcripts related to amino acid and lipid metabolism and transport that were present in these clusters, gene expression relevant to this type of CCNM and metabolite transport appears to be regulated by AS. GO categories involving other metabolic processes (listed in Figure 3) were not highly enriched, suggesting that, similarly to seed filling-related CCNM, these processes are also not globally regulated by AS.

AS has the potential to introduce or remove additional sequences to or from proteins that could result in truncated proteins or changes in protein localization, stability, interactions with other molecules, regulation, and/or biological function of the resulting proteins [14,15,16,17]. When considering CCNM and metabolite transport, only a few RNA isoforms showed completely different expression patterns, while the majority belonged to the same or similar clusters. Our focus here is on specific examples of isoforms showing different expression trends and representing diverse metabolic processes. Many novel transcripts that displayed strong differential expression when compared to the known transcripts contained a premature stop codon, and could encode only truncated, if any, peptides. The above-mentioned ACR12 gene encodes a plastidic amino acid transporter 1-like protein, and it was represented by two isoforms Glyma10g42580.5 and N6. While the first of these transcripts encodes a protein containing two ACT domains, the latter contains a premature stop codon.

Glycolytic 2,3-biphosphoglycerate-independent phosphoglycerate mutase-related isoform Glyma08g28530.1 (cluster 25) contains a metalloenzyme superfamily domain proposed to function in metal binding and catalysis. AS leads to the appearance of a premature stop codon in Glyma08g28530.N2 (cluster 45) and a substantial truncation of this domain. These two isoforms show completely different expression profiles. While Glyma08g28530.1 exhibits a gradual decrease throughout the time course, in contrast, the novel isoform shows an initial increase from day 5 to 20, followed by a decrease from day 30 to the end (Figure S2). In another example, Glyma04g14650.1 encodes a short acyl-CoA-binding protein containing overlapping acyl-CoA-binding and CoA-binding sites. This isoform displays a gradual slow decrease in transcript abundance (cluster 24). However, AS yielded a novel isoform Glyma04g14650.N2 (cluster 46) that does not appear to produce meaningful protein products, but peaks at day 30. Premature stop codons in transcripts lead to premature protein synthesis termination and, sometimes, subsequent peptide/protein degradation or NMD of such transcripts [18,19,20,21,22]. However, this remains to be confirmed on an individual basis along with the biological functions of these transcripts and resulting peptides.

However, in some instances, AS could lead to the production of large truncated proteins. Glyma20g32260.1 (cluster 56) encodes a full-length vacuolar amino acid transporter family protein with an intact amino acid permease domain SdaC, while this domain is truncated in Glyma20g32260.N3 (cluster 112) encoding the first half of the full-length protein. Only Glyma20g32260.1 shows an initial decrease in transcript levels from day 5 to 25 and both transcripts are expressed during the late maturation phases of soybean embryo development (Figure S2). With truncated proteins such as these, it is difficult to predict their function, but they have the potential to sequester substrates and cofactors, or interact with their full-length counterparts or other proteins and influence their activities.

**Figure 4:**
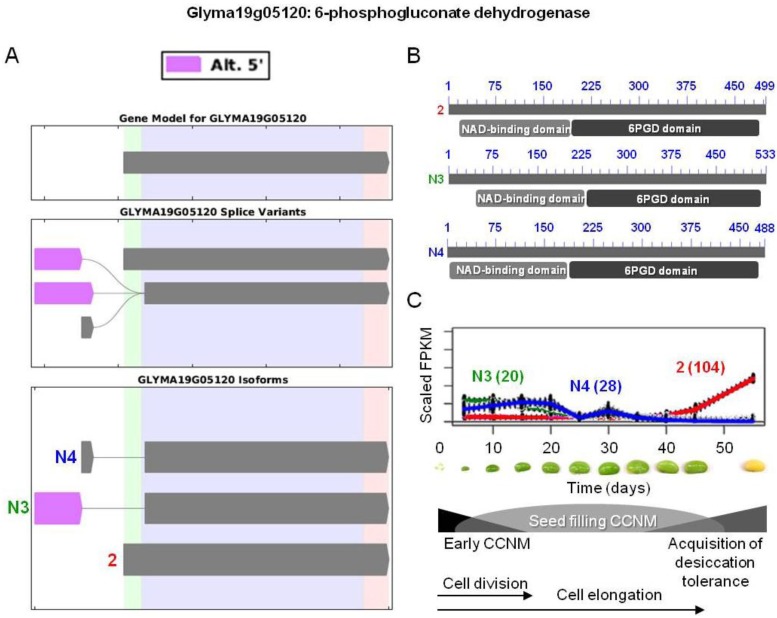
AS of 6-phosphogluconate dehydrogenase involved in oxidative pentose phosphate pathway. (**A**) SpliceGrapher representations of individual isoforms detected during soybean embryo development. Only Glyma19g05120.2, N3, and N4 (corresponding to 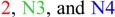) were differentially expressed. Alternative 5' splicing yielded these novel isoforms. (**B**) Proteins resulting from these three transcripts with the respective positions of NAD-binding domains (pfam03446) and 6-phosphogluconate dehydrogenase (6PGD) C-terminal domains (pfam00393). Domain-related information was obtained from NCBI [97]. Numbers in blue represent amino acid residues in the individual proteins. (**C**) Overlaid expression profiles of Glyma19g05120.2, N3, and N4, obtained from SplineCluster. Numbers shown in parenthesis represent the isoform clusters. Representative developing soybean embryos are shown for each time point along with a temporal representation of processes occurring during embryo development.

Three differentially expressed isoforms Glyma19g05120.2, N3, and N4 (clusters 104, 20, and 28; Figure 4) are predicted to encode a full-length 6-phosphogluconate dehydrogenase based on our results. This key enzyme in the oxidative pentose phosphate pathway is active in the chloroplast stroma during seed filling in soybean [4,5,6,7,8,9]. In this case, AS resulted in introduction of alternative start codons and subsequently potentially distinct sub-cellular localization of this enzyme. Based on the SoyBase database, the respective proteins are found in plastids, peroxisomes, and cytosol, though only the existence of the Glyma19g05120.2 isoform was known previously [69,71]. In the yeast *Candida albicans*, AS is known to be responsible for dual targeting of this enzyme to the cytosol and the peroxisomes [94]. The new soybean isoforms N3 and N4 could encode such differentially localized isoforms, with Glyma19g05120.N4 being cytosolic, as it lacks any transit peptide. In contrast, the subcellular location of the other two protein isoforms is unclear. Clusters 20 and 28 have somewhat similar trends, showing a moderate initial increase or steady expression during seed filling, followed by a subsequent decrease and low or no expression after day 35 (Figure 4). Based on this expression pattern, it is tempting to speculate that N3 is the plastidic isoform as it may be needed to provide ribulose-5P for the Rubisco bypass during seed filling [4,5,95]. Glyma19g05120.2 is expressed only during the late maturation and desiccation phases, starting at day 40, and could encode the peroxisomal isoform to generate NADPH for lipid degradation and antioxidant regeneration. 6-Phosphogluconate dehydrogenase was also found in pea peroxisomes and proposed to provide reductant for peroxisomal metabolism and recycling of oxidized ascorbate and glutathione [96] and this soybean isoform could have similar functions.

Phosphogluconate dehydrogenase is not the only example of an enzyme targeted to different compartments and originating from AS, but the roles of the resulting transcript isoforms for other genes are not apparent. Glyma02g47560.1-encoded LHCB2.1 (cluster 18) functions in PSII antennae and has a chloroplast targeting sequence. AS also yielded Glyma02g47560.2 (cluster 28) lacking the chloroplast targeting sequence. However, both proteins have an identical chlorophyll a/b binding domain, but only Glyma02g47560.1 is targeted to chloroplasts. Similarly, putative 5,10-methylene tetrahydrofolate dehydrogenase/cyclohydrolase predicted to function in folate interconversions originates from two isoforms having different transit peptides, Glyma09g39790.1 and N2 (clusters 15 and 50, respectively). The first transcript encodes a plastidic isoform of this enzyme, while the localization of the second one remains to be determined. Regardless of the localization, these isoforms show completely opposite transcription profiles. Glyma09g39790.1 displays an initial decrease in transcript levels from day 5 to 15, then a very low stable expression as opposed to Glyma09g39790.N2, which is only expressed during days 35 through 55 (Figure S2). Subcellular locations and functions of the proteins encoded by these new isoforms remain to be elucidated.

The most intriguing and rare scenario is when the resulting proteins contain or lack domains that have protein-protein interaction, catalytic, and/or known regulatory functions, as this could lead to proteins with potentially novel functions and regulatory capabilities. Such protein isoforms could have similar or completely different expression patterns, which, together with a systematic detailed domain analysis, can assist in dissecting their functions in specific processes. For example, if such protein isoforms have mutually exclusive expression patterns, they cannot physically interact. We were not able to identify any novel isoforms that would encode proteins with additional novel domains. However, random sampling of metabolic isoforms present in different clusters that showed similar trends revealed several genes where domains were modified such that they could have distinct catalytic, regulatory, or protein-protein interaction capabilities.

**Figure 5:**
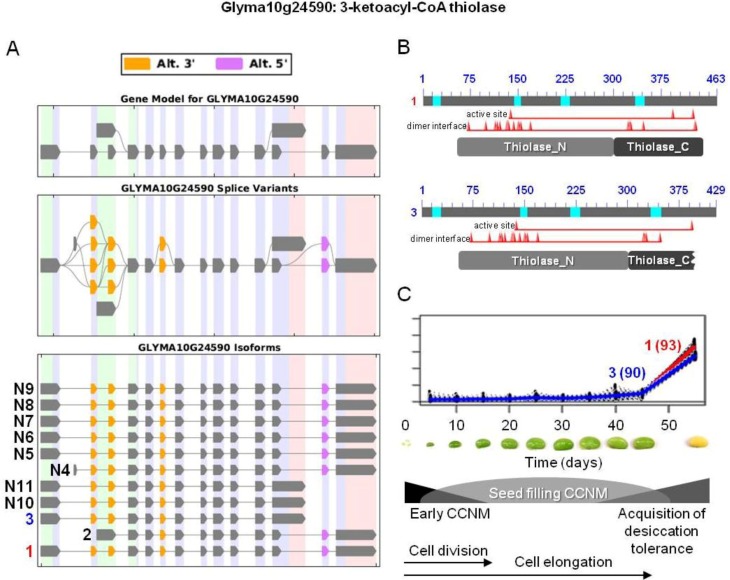
AS of 3-ketoacyl-CoA thiolase involved in fatty acid degradation during late maturation stages of soybean embryo development. (**A**) SpliceGrapher representations of individual isoforms detected during soybean embryo development. Alternative 3' and/or 5' splicing of many novel isoforms were identified. Glyma10g24590.1 and 3 correspond to 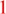 and 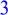 were the only two transcripts displaying differential expression. (**B**) Proteins resulting from two isoforms of interest with the respective positions of active and dimer interface sites in the *N*- and *C*-terminal thiolase domains (pfam00108 and pfam02803, respectively), showing the positions of individual amino acid residues (red triangles). Bright blue sections of the protein show the sequences that were not included in domain analysis due to an amino acid composition-related bias. (**C**) Overlaid expression profiles of Glyma10g24590.1 and 3 obtained from SplineCluster.

In soybean, 3-ketoacyl-CoA thiolase 3 is a fatty acid β-oxidation enzyme encoded by isoforms Glyma10g24590.1, 2, and 3. Isoforms Glyma10g24590.1 and 3 encode these enzymes with alternate C-termini possessing the acetyl-CoA C-acyltransferase multidomain containing specific sites (Figure 5). Glyma10g24590.1 (cluster 93) has all expected residues in the thiolase active and dimer interface sites. In contrast, Glyma10g24590.3 (cluster 90) is missing one of three of the amino acid residues found in thiolases and two of twenty of the residues that make up the dimer interface domain. In Arabidopsis, the closest homolog of Glyma10g24590 is At2g33150, a peroxisomal 3-ketoacyl-CoA thiolase, but another close Arabidopsis homolog At5g48880 is also subjected to AS yielding both a peroxisomal and cytosolic isoforms [45]. The At2g33150-encoded enzyme is involved in seed dormancy and germination, as well as turnover of fatty acids during natural and dark-induced senescence [98,99,100]. This enzyme also plays an important role in positive regulation of ABA signaling by acting downstream of a WRKY TF involved in ABA-mediated signaling, thus promoting the embryonic nature of developing embryos and suppressing germination-related processes in Arabidopsis [101]. This potential and unexpected dual role for this enzyme as a component of ABA signaling provides a connection between CCNM and acquisition of desiccation tolerance/dormancy acquisition processes during late maturation phases of oilseed embryo development.

#### 3.2.2. AS of Splicing-Associated Transcripts, Acquisition of Dormancy and Desiccation Tolerance, ABA, and Other Phytohormone-Related Events

In Figure S3, a summary of GO enrichment results for categories related to dormancy acquisition and desiccation tolerance are shown, together with an indication of the clusters responsible for that enrichment. Interestingly, clusters reflecting splicing events early on in the time course of seed development contained isoforms of genes associated with seed dormancy. In particular, in clusters 9, 10, 15, and 21, isoforms of Arabidopsis homologs of regulatory genes (Glyma08g19650.2, soybean homolog of separase) were differentially expressed during this first phase of seed development, corresponding to the cell division phase, as shown in Figure S3. Isoforms of four transcripts, whose homologs in Arabidopsis are EMB genes (“embryo lethal”, EMB2656, 1968, and 2271) appeared in the clusters corresponding to early events in seed development. Clusters 60 and 85, corresponding to the late phase of seed development involving acquisition of dormancy and desiccation tolerance, were also enriched for the GO category “seed dormancy process”, albeit with fewer genes overall. Mutant studies using the specific isoforms, which were differentially expressed over the time course, are needed to determine if, and how, these isoforms influence dormancy acquisition. In the case of, for example, Glyma05g35280.3, whose Arabidopsis homolog is At3g24440 (VRN7) encoding a fibronectin, type III protein associated with chromatin modification and epigenetic effects, it would be interesting to identify its interacting partners, and whether only the third known isoform influences subsequent developmental processes. The same holds for Glyma01g02350.3, of which the Arabidopsis homolog is IAA8, a gene associated with the regulation of organ formation. It is important to note that the information provided by GO comes from transcriptional studies, and no extensive reservoir of information on isoforms is yet available. Thus, rigorous associations between developmental processes and splicing events have not yet been made, in the vast majority of cases. Nonetheless, the appearance, early on in seed development, of transcripts corresponding to genes encoding regulators of organ formation, suggests a particular role for those isoforms in pattern embryo formation.

GO categories associated with response to ABA were enriched in multiple clusters from cluster 48 onwards. All clusters that were enriched for ABA responses show a common pattern, with increases in differential expression towards the late stages of seed development. This is, perhaps, to be expected, as ABA is well known as the phytohormone that confers dormancy on maturing seeds [10,11,12,13]. However, it is noteworthy that this response develops, not during the final stages of maturation, but much earlier, during seed filling stages. This result suggests that the dormancy induction process may be initiated on a different time scale than was previously thought. In keeping with this inference, the GO category “maintenance of dormancy” was enriched for differentially expressed isoforms in clusters 63 and 67, rather than the clusters displaying a sharp rise at the far end of development, when dormancy acquisition has already occurred.

**Figure 6:**
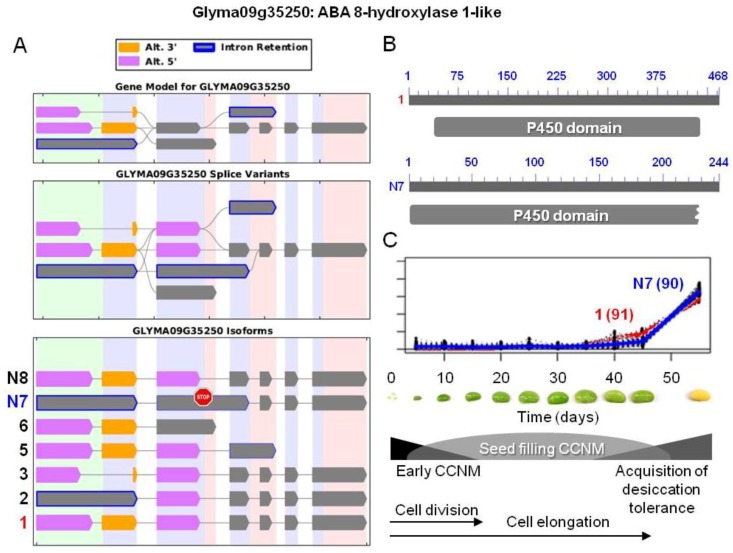
AS of ABA 8'-hydroxylase 1-like protein possibly involved in ABA degradation. (**A**) SpliceGrapher representations of individual isoforms detected during soybean embryo development. Alternative 3' and/or 5' splicing, and/or intron retention was observed in these isoforms. Glyma10g24590.1 and N7 corresponded to isoforms 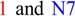. The stop sign in N7 indicates a premature stop codon. (**B**) Proteins resulting from the two isoforms of interest, showing the respective positions of the p450 domains (pfam00067). Isoform N7 contains a truncated p450 domain. (**C**) Overlaid transcriptional profiles of Glyma10g24590.1 and N7 obtained from SplineCluster.

Two unexpected GO categories “release of seed from dormancy” and “exit from dormancy” were enriched for differential splicing in a number of clusters from 52 onwards. Genes encoding ABA 8'-hydroxlyase, a member of the cytochrome P450 gene family and an ABA-degrading enzyme, and ABA 8'-hydroxylase-like proteins were over-represented in these clusters. On inspection, it appears that several isoforms among the group corresponded to full-length P450 proteins, and, hence, it would be expected that they are enzymatically active. However, some isoforms lacked a portion of the P450 domain due to a premature stop codon (Figure 6). Since ABA levels are maintained as dormancy is acquired, it seems unlikely that ABA degradation takes place during that time period. It is possible that the group of transcripts encoding these ABA-degrading enzymes is stored in either P-bodies or stress granules as we proposed for photosynthesis-related transcripts, to be translated upon imbibition and the initiation of germination.

**Figure 7:**
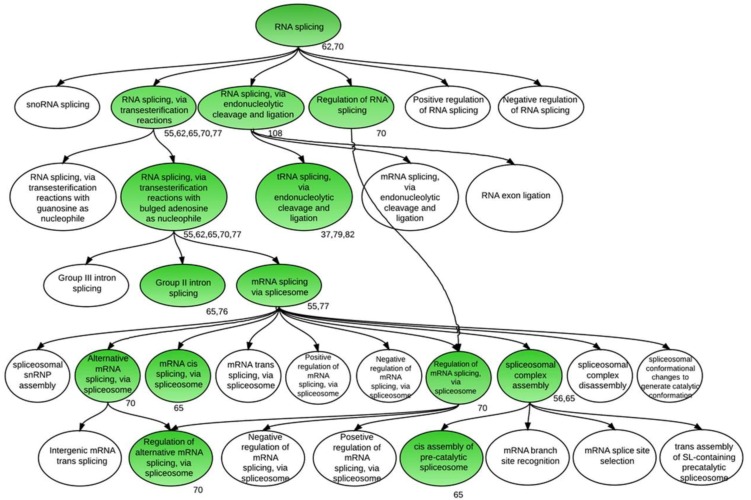
Tree displaying GO-enriched processes regulated by AS and associated with RNA splicing. Filtered enriched GO categories were searched for three keywords (“RNA splicing”, “dormancy”, and “response to hormone stimulus”) in all 114 clusters and the GO database [66] was used to identify the parents and children for each GO term. These were then combined to obtain a single tree representing all enriched GO terms corresponding to each keyword. This large tree was then separated into three sub-trees corresponding to these three processes and the tree corresponding to the “RNA splicing” category is shown. GO terms that were enriched significantly (*p* < 0.05) in genes involved in RNA splicing are shown in green. The clusters containing these RNA-splicing-related genes are shown below the corresponding category. This figure was generated in LucidChart [106].

Figure 7 shows a GO tree corresponding to enrichment of terms related to splicing. All clusters that showed enrichment for splicing-associated processes for mRNA fell between clusters 55 and 82, corresponding, as in the case of the ABA-associated clusters, to the late phases of seed development. It is known that the splicing process itself is affected by abiotic stress [102,103], and the acquisition of desiccation tolerance in seeds shares many gene expression events with stress responses reported in vegetative tissue [104]. Much AS, associated with splicing factors, occurred in cluster 70 (Figure S2; Table S3), including the appearance of specific isoforms of the soybean homologs of SR34a (a Ser/Arg-rich protein known to be involved in splicing, Glyma06g14060.2). RRC1, an RNA recognition motif (RRM)-containing protein, Glyma08g34030.4, as well as Glyma04g07300.3, a homolog of At4g32420 encoding a cyclophilin-like peptidyl-prolyl *cis*-*trans* isomerase family protein known to interact with SR proteins in pre-splicing events at the spliceosome [105], were also present in cluster 70.

The established interconnectedness of seed signaling pathways involving multiple hormones is reflected in the AS events depicted in Figure S4. In addition to expected categories associated with ABA-mediated signaling, some differential splicing occurred in categories related to each of the major hormone classes. Jasmonic acid and ABA are well established as regulators of seed development, including through epigenetic mechanisms [107], as is ethylene (interacts with ABA [108]), auxin (also interacting with ABA [73]), brassinosteroids [109], and gibberellins (in a negative interaction with ABA [72]). In the case of auxin, recent results have shown that auxin-mediated signaling acts to regulate the expression of ABI3, the principal regulator of seed dormancy [73]. Evidence for differential splicing among transcripts related to auxin signaling is shown in Figure S4 and Table S3, and it is interesting that these events occur mostly relatively early on in the soybean embryo developmental time course, in clusters 9 through 58, with two parent categories enriched in clusters 77 and 85. Some of the RNA processing activities associated with auxin signaling that are presented in Figure S4 may be a reflection of the formation of primordial organs in the developing seed. It will also be interesting to use this information as a starting point to identify further links between auxin signaling and ABA-mediated dormancy.

## 4. Conclusions

Seed filling and acquisition of desiccation tolerance and dormancy in maturing oilseed embryos represent important developmental stages crucial for acquiring and maintaining seed viability. AS plays roles in post-transcriptional regulation of developmental, metabolic, and stress-related processes in plants. Bioinformatics analyses enabled identification and global analyses of various AS events in developing soybean embryos. As expected, many pre-mRNAs encoding enzymes and proteins involved in diverse aspects of CCNM and signaling pathways of desiccation-related processes as well as components of the actual splicing machinery were subjected to AS. Late maturation stages of soybean embryo development were characterized as having a larger proportion of AS-derived isoforms than other stages, which can be explained by induction of splicing as part of maturation and desiccation tolerance acquisition pathways. These pathways involve stress-like hormonal responses and signaling pathways known to be regulated at the AS level. Detailed analyses of selected isoforms involved in CCNM and ABA-related metabolism revealed possible roles for AS in regulating activities, subcellular localization, and protein-protein interactions of the resulting proteins. These are just first steps in the comprehensive analysis of the large datasets generated in this study that will provide a vast resource for further data mining and testable hypothesis generation. 

## Supplementary Files

Supplementary File 1 Supplementary Figure S1 (PDF, 112 KB)

Supplementary File 2 Supplementary Figure S2 (PDF, 1478 KB)

Supplementary File 3 Supplementary Figure S3 (DOCX, 157 KB)

Supplementary File 4 Supplementary Figure S4 (DOCX, 140 KB)

Supplementary File 5 Supplementary Table S1 (XLSX, 11 KB)

Supplementary File 6 Supplementary Table S2 (XLSX, 11523 KB)

Supplementary File 7 Supplementary Table S3 (XLSX, 693 KB)

Supplementary File 8 merged (ZIP, 16035 KB)
