# Supplementary material for: Changes in RNA Splicing in Developing Soybean (Glycine max) Embryos
Source: Biology (Basel). 2013 Nov 21;2(4):1311–37. doi: 10.3390/biology2041311 (PMC4009788; doi:10.3390/biology2041311)
Supplement: Supplementary File 1 — Supplementary Figure S1 (PDF, 112 KB) [file biology-02-01311-s001.pdf]

**Figure S1.** Validation of relative expression of gene and isoform expression obtained from RNA-seq by qPCR. RNA-seq and qPCR analyses were performed as described in the Experimental Section. Relative (qPCR) and FPKM (RNA-seq) expression values are presented as an average ± SD of three biological replicates.

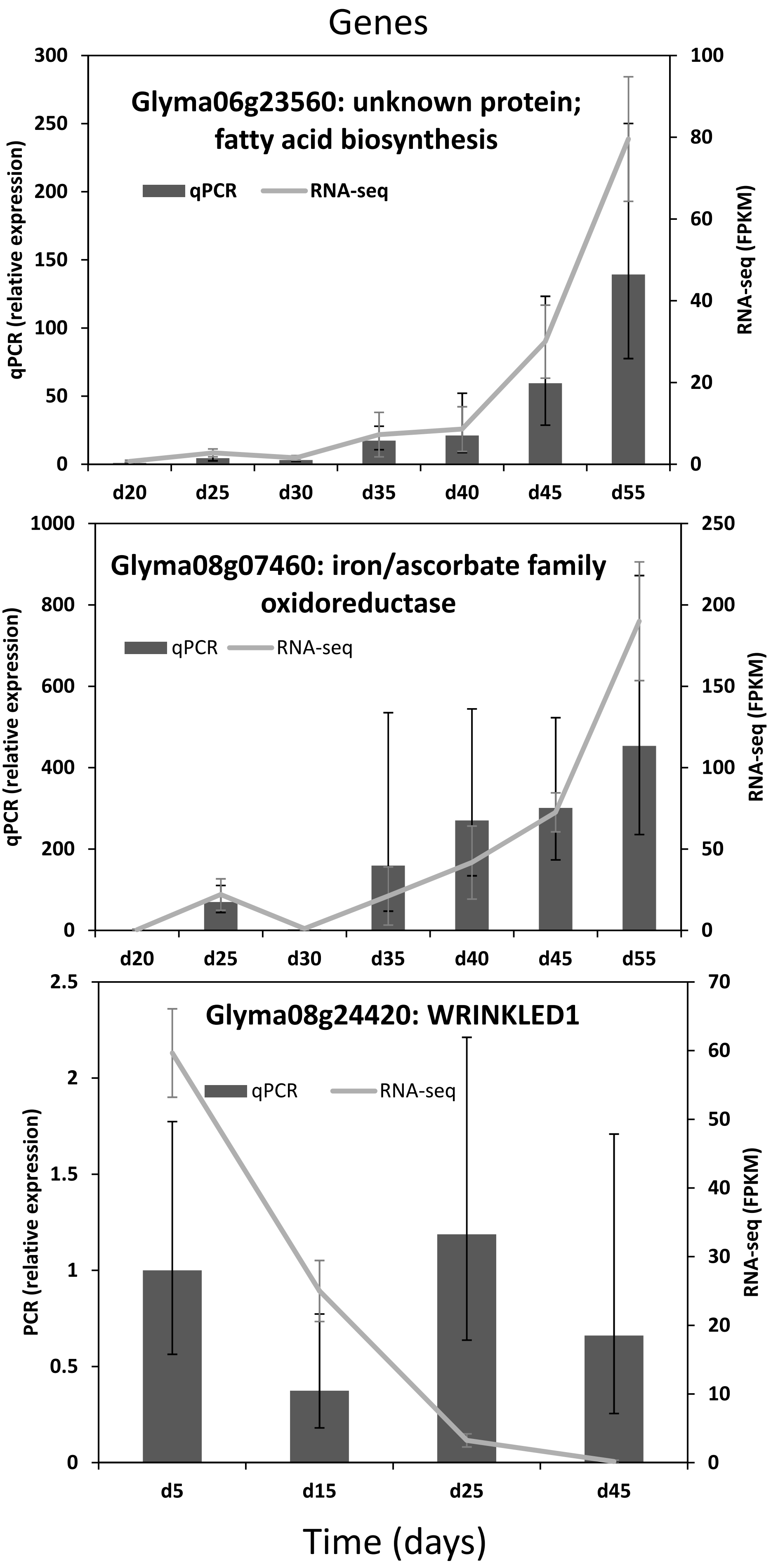
Genes
Glyma06g23560: unknown protein; fatty acid biosynthesis
qPCR
RNA-seq
qPCR (relative expression)
RNA-seq (FPKM)
0
50
100
150
200
250
300
0
20
40
60
80
100
d20
d25
d30
d35
d40
d45
d55
Glyma08g07460: iron/ascorbate family oxidoreductase
qPCR
RNA-seq
qPCR (relative expression)
RNA-seq (FPKM)
0
200
400
600
800
1000
0
50
100
150
200
250
d20
d25
d30
d35
d40
d45
d55
Glyma08g24420: WRINKLED1
qPCR
RNA-seq
PCR (relative expression)
RNA-seq (FPKM)
0
0.5
1
1.5
2
2.5
0
10
20
30
40
50
60
70
d5
d15
d25
d45
Time (days)

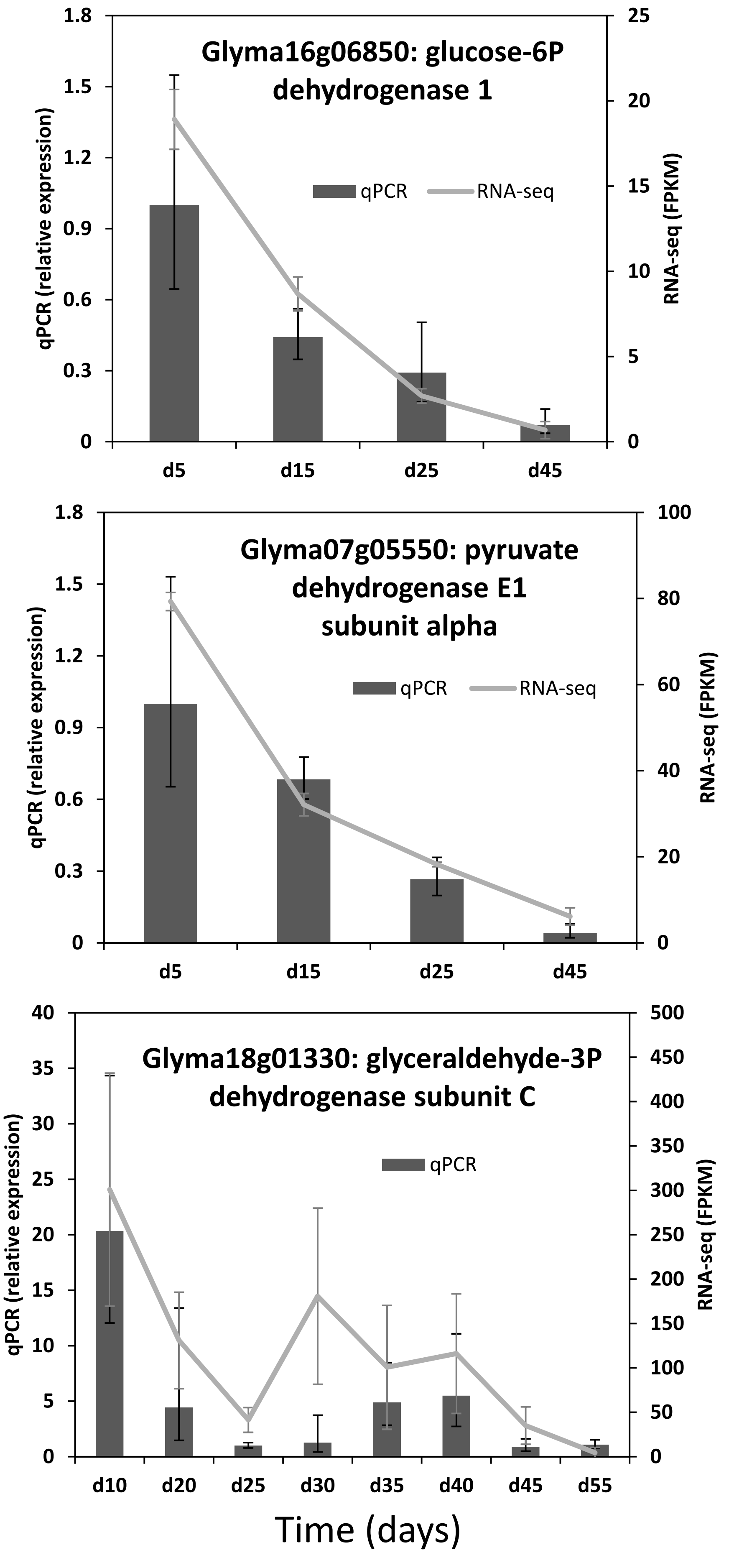
Glyma16g06850: glucose-6P dehydrogenase 1
qPCR
RNA-seq
qPCR (relative expression)
RNA-seq (FPKM)
0
0.3
0.6
0.9
1.2
1.5
1.8
0
5
10
15
20
25
d5
d15
d25
d45
Glyma07g05550: pyruvate dehydrogenase E1 subunit alpha
qPCR
RNA-seq
qPCR (relative expression)
RNA-seq (FPKM)
0
0.3
0.6
0.9
1.2
1.5
1.8
0
20
40
60
80
100
d5
d15
d25
d45
Glyma18g01330: glyceraldehyde-3P dehydrogenase subunit C
qPCR
qPCR (relative expression)
RNA-seq (FPKM)
0
5
10
15
20
25
30
35
40
0
50
100
150
200
250
300
350
400
450
500
d10
d20
d25
d30
d35
d40
d45
d55
Time (days)

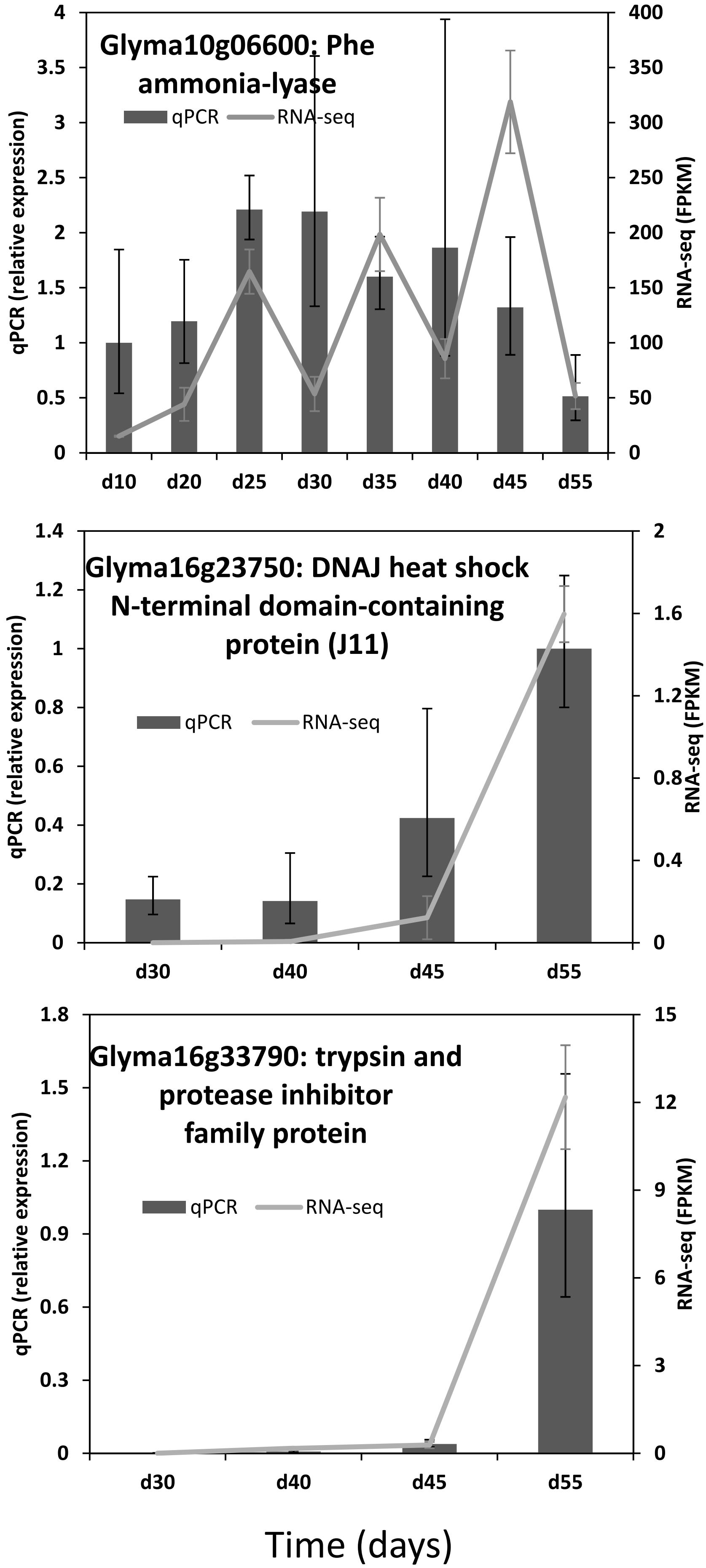

Glyma10g06600: Phe ammonia-lyase
qPCR
RNA-seq
qPCR (relative expression)
RNA-seq (FPKM)
0
0.5
1
1.5
2
2.5
3
3.5
4
0
50
100
150
200
250
300
350
400
d10
d20
d25
d30
d35
d40
d45
d55
Glyma16g23750: DNAJ heat shock N-terminal domain-containing protein (J11)
qPCR
RNA-seq
qPCR (relative expression)
RNA-seq (FPKM)
0
0.2
0.4
0.6
0.8
1
1.2
1.4
0
0.4
0.8
1.2
1.6
2
d30
d40
d45
d55
Glyma16g33790: trypsin and protease inhibitor family protein
qPCR
RNA-seq
qPCR (relative expression)
RNA-seq (FPKM)
0
0.3
0.6
0.9
1.2
1.5
1.8
0
3
6
9
12
15
d30
d40
d45
d55
Time (days)

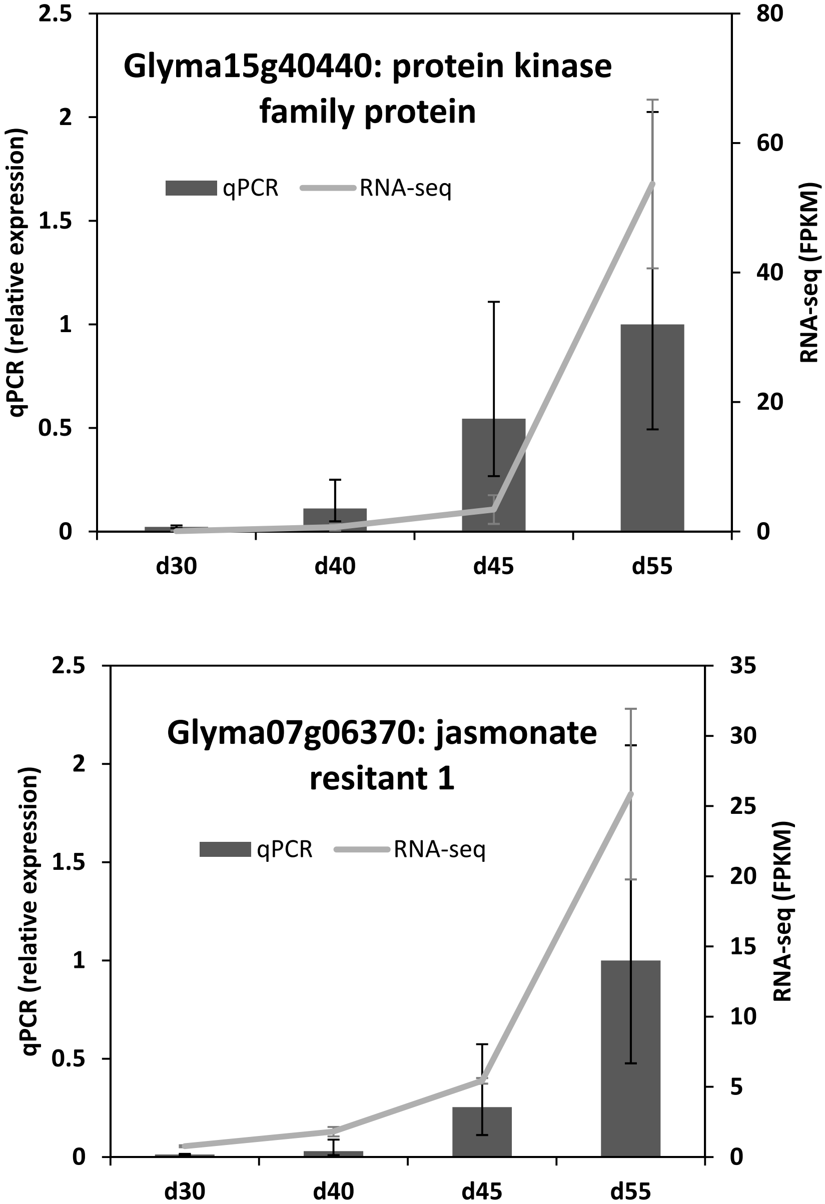
Glyma15g40440: protein kinase family protein
qPCR
RNA-seq
qPCR (relative expression)
RNA-seq (FPKM)
2.5
2
1.5
1
0.5
0
80
60
40
20
0
d30
d40
d45
d55
Glyma07g06370: jasmonate resitant 1
qPCR
RNA-seq
qPCR (relative expression)
RNA-seq (FPKM)
2.5
2
1.5
1
0.5
0
35
30
25
20
15
10
5
0
d30
d40
d45
d55

Time (days)

# Splice variants

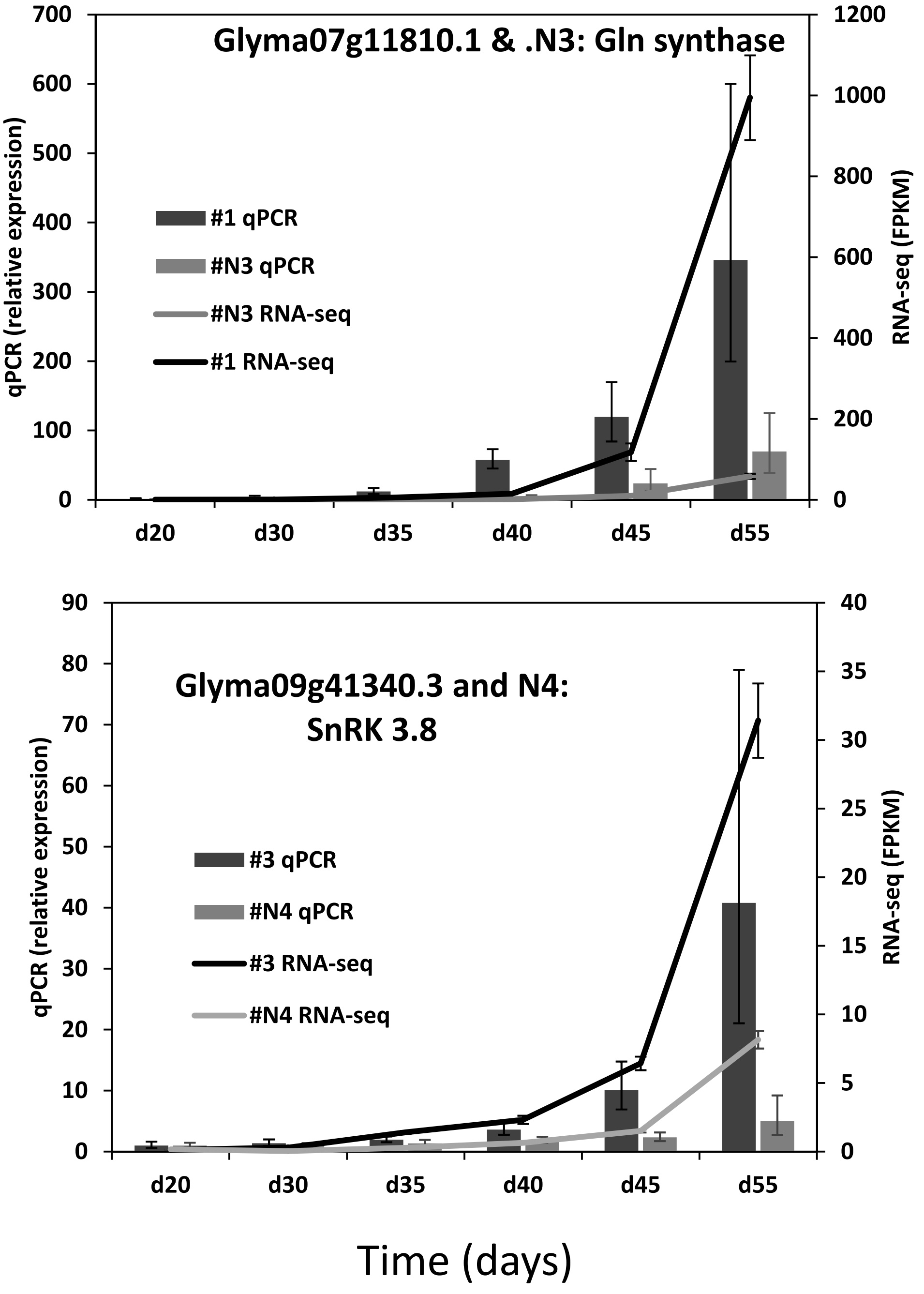

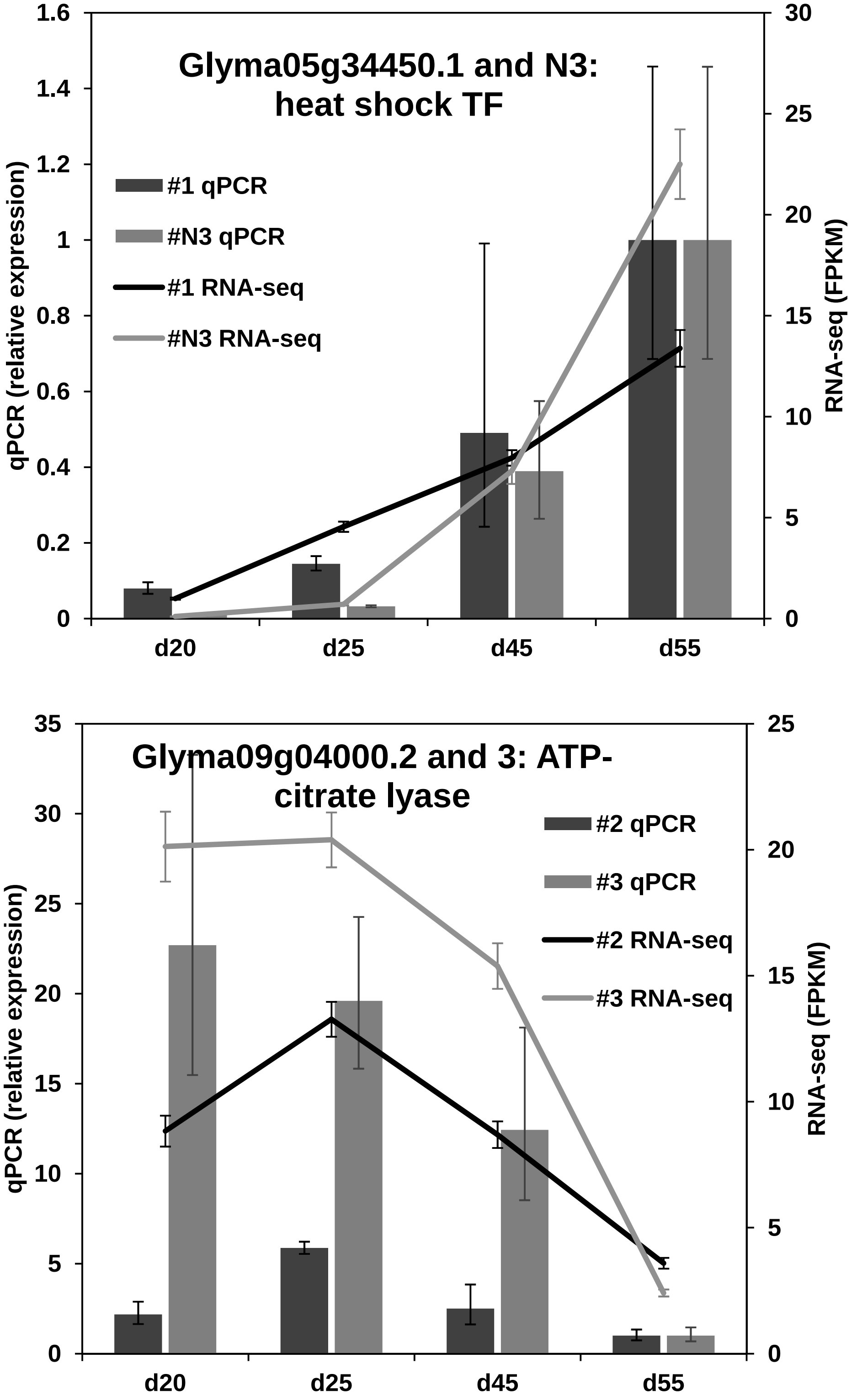
Glyma05g34450.1 and N3: heat shock TF
#1 qPCR
#N3 qPCR
#1 RNA-seq
#N3 RNA-seq
qPCR (relative expression)
RNA-seq (FPKM)
0
0.2
0.4
0.6
0.8
1
1.2
1.4
1.6
0
5
10
15
20
25
30
d20
d25
d45
d55
Glyma09g04000.2 and 3: ATP-citrate lyase
#2 qPCR
#3 qPCR
#2 RNA-seq
#3 RNA-seq
qPCR (relative expression)
RNA-seq (FPKM)
0
5
10
15
20
25
30
35
0
5
10
15
20
25
d20
d25
d45
d55

Time (days)
