# Supplementary material for: Changes in RNA Splicing in Developing Soybean (Glycine max) Embryos
Source: Biology (Basel). 2013 Nov 21;2(4):1311–37. doi: 10.3390/biology2041311 (PMC4009788; doi:10.3390/biology2041311)
Supplement: Supplementary File 2 — Supplementary Figure S2 (PDF, 1478 KB) [file biology-02-01311-s002.pdf]

Figure S2. Co-clustering analysis of 15368 transcripts displaying temporal differential expression during soybean embryo development. Co-clustering analysis was performed by using SplineCluster, as described in the Experimental Section, and resulted in 114 clusters.

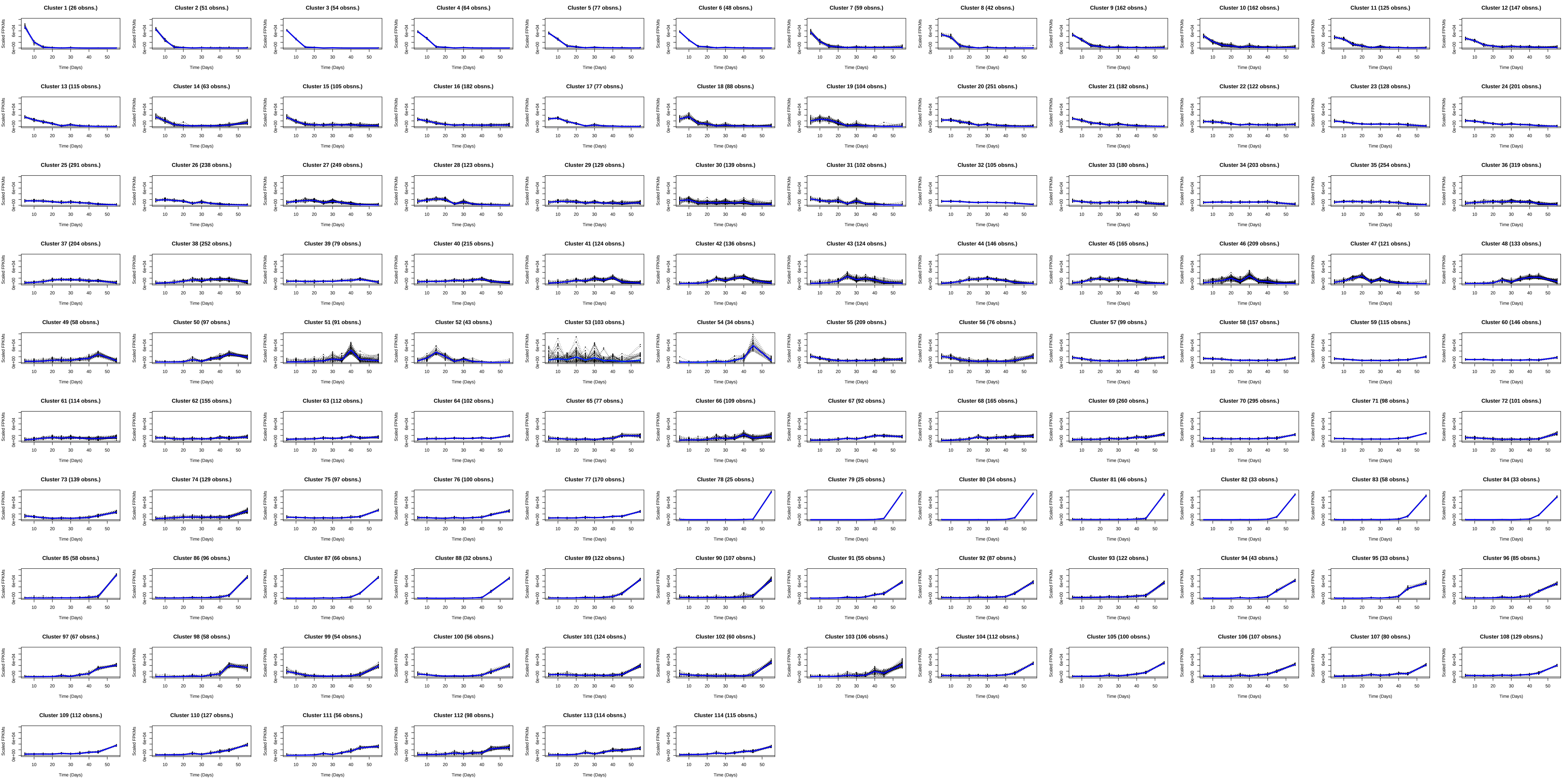
Cluster 1 (26 obsns.)
Cluster 2 (51 obsns.)
Cluster 3 (54 obsns.)
Cluster 4 (64 obsns.)
Cluster 5 (77 obsns.)
Cluster 6 (48 obsns.)
Cluster 7 (59 obsns.)
Cluster 8 (42 obsns.)
Cluster 9 (162 obsns.)
Cluster 10 (162 obsns.)
Cluster 11 (125 obsns.)
Cluster 12 (147 obsns.)
Cluster 13 (115 obsns.)
Cluster 14 (63 obsns.)
Cluster 15 (105 obsns.)
Cluster 16 (182 obsns.)
Cluster 17 (77 obsns.)
Cluster 18 (88 obsns.)
Cluster 19 (104 obsns.)
Cluster 20 (251 obsns.)
Cluster 21 (182 obsns.)
Cluster 22 (122 obsns.)
Cluster 23 (128 obsns.)
Cluster 24 (201 obsns.)
Cluster 25 (291 obsns.)
Cluster 26 (238 obsns.)
Cluster 27 (249 obsns.)
Cluster 28 (123 obsns.)
Cluster 29 (129 obsns.)
Cluster 30 (139 obsns.)
Cluster 31 (102 obsns.)
Cluster 32 (105 obsns.)
Cluster 33 (180 obsns.)
Cluster 34 (203 obsns.)
Cluster 35 (254 obsns.)
Cluster 36 (319 obsns.)
Cluster 37 (204 obsns.)
Cluster 38 (252 obsns.)
Cluster 39 (79 obsns.)
Cluster 40 (215 obsns.)
Cluster 41 (124 obsns.)
Cluster 42 (136 obsns.)
Cluster 43 (124 obsns.)
Cluster 44 (146 obsns.)
Cluster 45 (165 obsns.)
Cluster 46 (209 obsns.)
Cluster 47 (121 obsns.)
Cluster 48 (133 obsns.)
Cluster 49 (58 obsns.)
Cluster 50 (97 obsns.)
Cluster 51 (91 obsns.)
Cluster 52 (43 obsns.)
Cluster 53 (103 obsns.)
Cluster 54 (34 obsns.)
Cluster 55 (209 obsns.)
Cluster 56 (76 obsns.)
Cluster 57 (99 obsns.)
Cluster 58 (157 obsns.)
Cluster 59 (115 obsns.)
Cluster 60 (146 obsns.)
Cluster 61 (114 obsns.)
Cluster 62 (155 obsns.)
Cluster 63 (112 obsns.)
Cluster 64 (102 obsns.)
Cluster 65 (77 obsns.)
Cluster 66 (109 obsns.)
Cluster 67 (92 obsns.)
Cluster 68 (165 obsns.)
Cluster 69 (260 obsns.)
Cluster 70 (295 obsns.)
Cluster 71 (98 obsns.)
Cluster 72 (101 obsns.)
Cluster 73 (139 obsns.)
Cluster 74 (129 obsns.)
Cluster 75 (97 obsns.)
Cluster 76 (100 obsns.)
Cluster 77 (170 obsns.)
Cluster 78 (25 obsns.)
Cluster 79 (25 obsns.)
Cluster 80 (34 obsns.)
Cluster 81 (46 obsns.)
Cluster 82 (33 obsns.)
Cluster 83 (58 obsns.)
Cluster 84 (33 obsns.)
Cluster 85 (58 obsns.)
Cluster 86 (96 obsns.)
Cluster 87 (66 obsns.)
Cluster 88 (32 obsns.)
Cluster 89 (122 obsns.)
Cluster 90 (107 obsns.)
Cluster 91 (55 obsns.)
Cluster 92 (87 obsns.)
Cluster 93 (122 obsns.)
Cluster 94 (43 obsns.)
Cluster 95 (33 obsns.)
Cluster 96 (85 obsns.)
Cluster 97 (67 obsns.)
Cluster 98 (58 obsns.)
Cluster 99 (54 obsns.)
Cluster 100 (56 obsns.)
Cluster 101 (124 obsns.)
Cluster 102 (60 obsns.)
Cluster 103 (106 obsns.)
Cluster 104 (112 obsns.)
Cluster 105 (100 obsns.)
Cluster 106 (107 obsns.)
Cluster 107 (80 obsns.)
Cluster 108 (129 obsns.)
Cluster 109 (112 obsns.)
Cluster 110 (127 obsns.)
Cluster 111 (56 obsns.)
Cluster 112 (98 obsns.)
Cluster 113 (114 obsns.)
Cluster 114 (115 obsns.)
Scaled FPKMs
0e+00
6e+04
Time (Days)
10
20
30
40
50
