# Supplementary material for: Changes in RNA Splicing in Developing Soybean (Glycine max) Embryos
Source: Biology (Basel). 2013 Nov 21;2(4):1311–37. doi: 10.3390/biology2041311 (PMC4009788; doi:10.3390/biology2041311)

**Figure S3.** GO enrichment tree depicting processes regulated by AS and associated with dormancy. This figure was generated as described in Experimental Section and the legend of Figure 7. The sub-tree relevant to “dormancy” is shown. GO terms that were enriched significantly in genes involved in dormancy are shown in green along with the corresponding clusters containing these dormancy-related genes. The hatched “response to abscisic acid stimulus” category is shown as a separate sub-tree in Supplementary Figure S4.


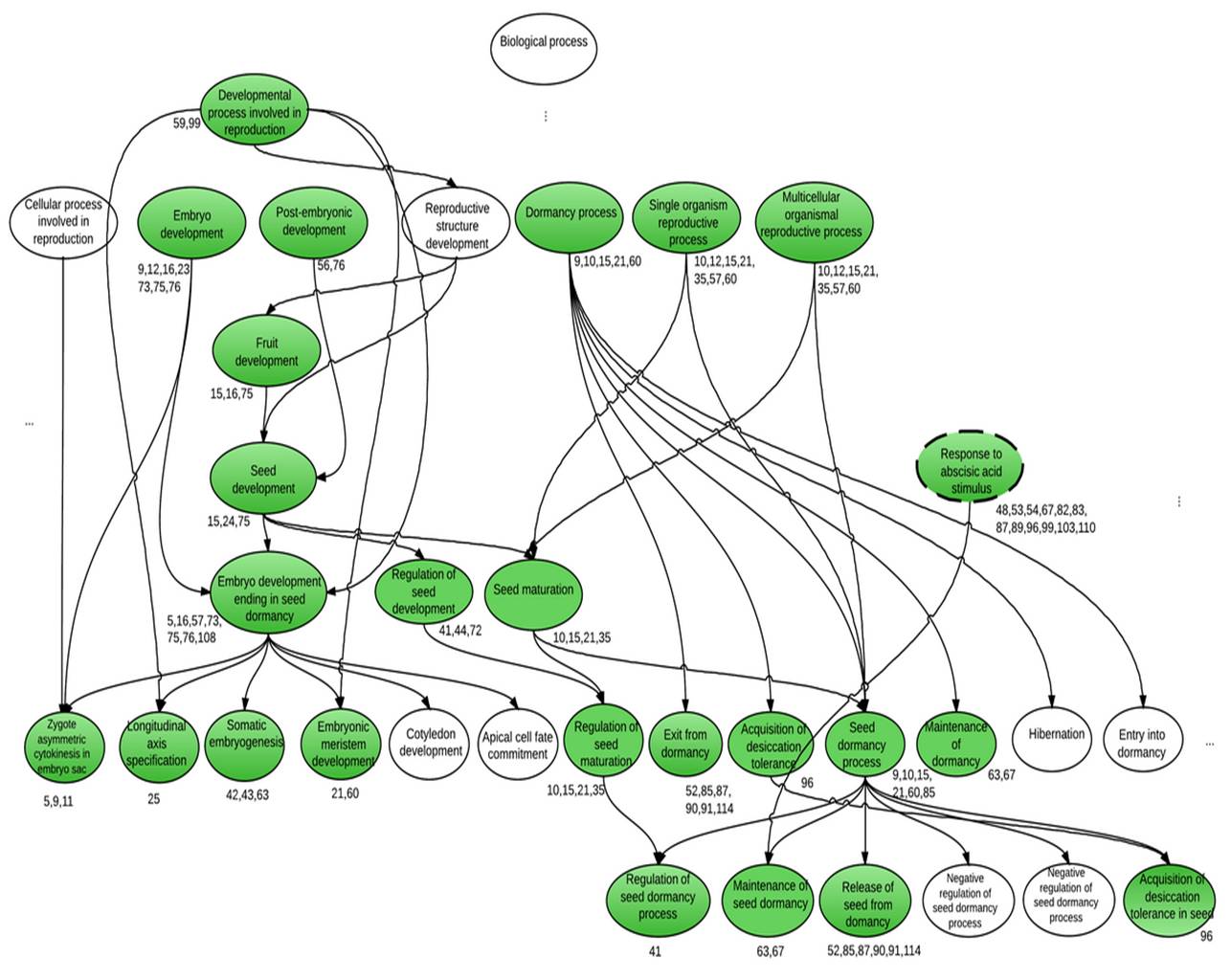

Supplement: Supplementary File 3 — Supplementary Figure S3 (DOCX, 157 KB) [file biology-02-01311-s003.docx]
