# Supplementary material for: Changes in RNA Splicing in Developing Soybean (Glycine max) Embryos
Source: Biology (Basel). 2013 Nov 21;2(4):1311–37. doi: 10.3390/biology2041311 (PMC4009788; doi:10.3390/biology2041311)

**Figure S4.** GO enrichment tree showing hormone-related processes regulated by AS over the time course of seed development. The hatched “maintenance of seed dormancy” category is displayed as an individual sub-tree in Supplementary Figure S3. This figure was generated as described in Figure 7.


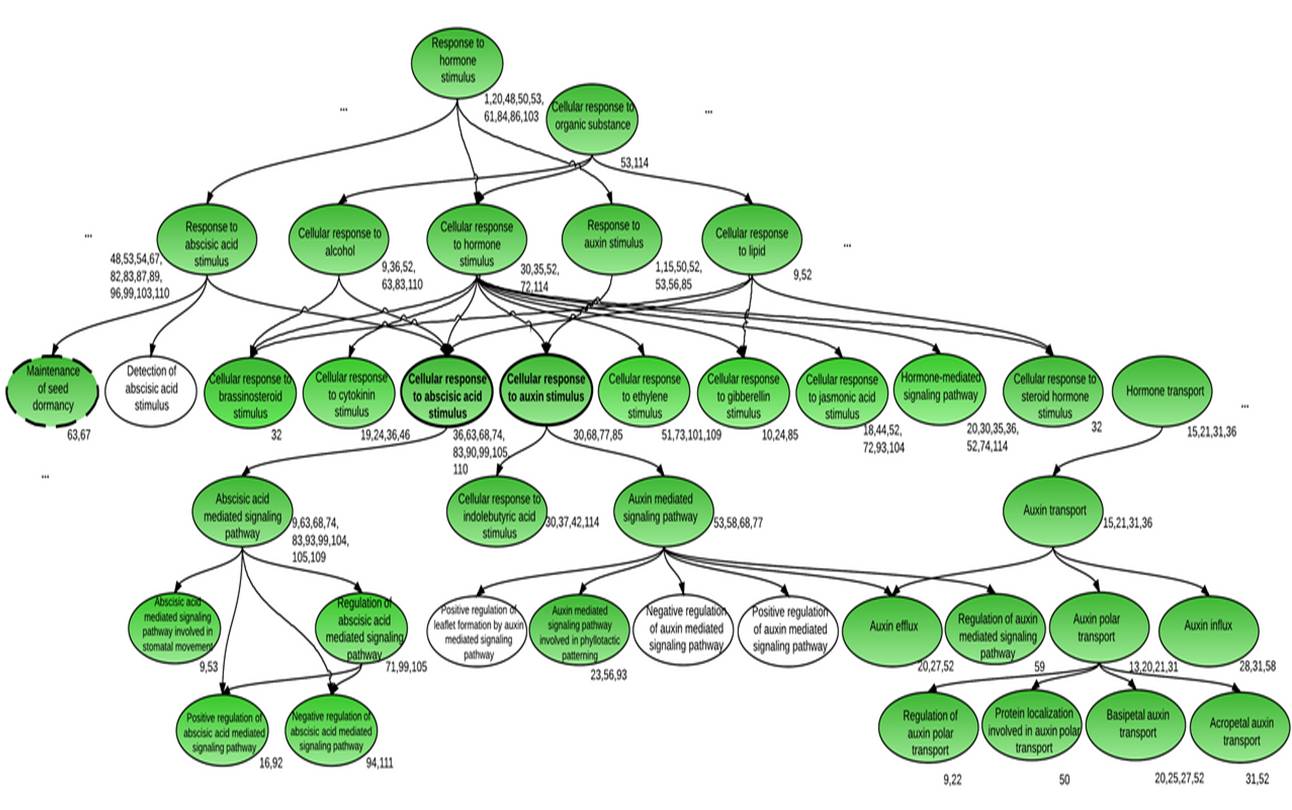

Supplement: Supplementary File 4 — Supplementary Figure S4 (DOCX, 140 KB) [file biology-02-01311-s004.docx]
